# Laparoscopic approach of inguinal hernia associated with adult cryptorchidism: case series and literature review

**DOI:** 10.1093/jscr/rjae232

**Published:** 2024-04-08

**Authors:** Claudiu Ungureanu, Floris Stanculea, Octav Ginghina, Daniel A Cristian, Valentin T Grigorean, Razvan Popescu, Dragos Georgescu, Niculae Iordache

**Affiliations:** “Carol Davila” University of Medicine and Pharmacy, 37, Dionisie Lupu Street, 020021 Bucharest, Romania; General Surgery Department, “Sf. Ioan” Clinical Emergency Hospital, 13, Vitan Barzesti Street, 042122 Bucharest, Romania; “Carol Davila” University of Medicine and Pharmacy, 37, Dionisie Lupu Street, 020021 Bucharest, Romania; General Surgery Department, “Sf. Ioan” Clinical Emergency Hospital, 13, Vitan Barzesti Street, 042122 Bucharest, Romania; “Carol Davila” University of Medicine and Pharmacy, 37, Dionisie Lupu Street, 020021 Bucharest, Romania; General Surgery Department, “Prof. Dr. Alexandru Trestioreanu” Oncological Institute, 252, Fundeni Street, 022328 Bucharest, Romania; “Carol Davila” University of Medicine and Pharmacy, 37, Dionisie Lupu Street, 020021 Bucharest, Romania; General Surgery Department, “Coltea” Clinical Hospital, 1, I.C.Bratianu Street, 030171 Bucharest, Romania; “Carol Davila” University of Medicine and Pharmacy, 37, Dionisie Lupu Street, 020021 Bucharest, Romania; General Surgery Department, “Bagdasar-Arseni” Clinical Emergency Hospital, 12, Berceni Street, 041915 Bucharest, Romania; “Carol Davila” University of Medicine and Pharmacy, 37, Dionisie Lupu Street, 020021 Bucharest, Romania; Urology Department, “Th.Burghele” Clinical Hospital, 20, Panduri Street, 050659 Bucharest, Romania; “Carol Davila” University of Medicine and Pharmacy, 37, Dionisie Lupu Street, 020021 Bucharest, Romania; General Surgery Department, “Dr. I. Cantacuzino” Clinical Hospital, 5-7, Ioan-Movila Street, 022904 Bucharest, Romania; “Carol Davila” University of Medicine and Pharmacy, 37, Dionisie Lupu Street, 020021 Bucharest, Romania; General Surgery Department, “Sf. Ioan” Clinical Emergency Hospital, 13, Vitan Barzesti Street, 042122 Bucharest, Romania

**Keywords:** hernia, cryptorchidism, laparoscopic, Inguinal hernia, transabdominal preperitoneal repair (TEP), ectopic testicle, trans-abdominal preperitoneal (TAPP)

## Abstract

Cryptorchidism is defined as the extra-scrotal position of the testes. It is a common disorder in male children, but rarely in adult patients. The association of cryptorchidism with hernia is a common finding in childhood, but is not frequent in adults or the elderly. Herein, we report a series of three cases (28-, 24-, and 34-year-old men) of adult inguinal hernia combined with cryptorchidism successfully managed by laparoscopic surgery under the same operative view. Laparoscopic transabdominal preperitoneal repair and orchiectomy were performed in all patients. No complications occurred in the postoperative period, and the patients were discharged on the first or second postoperative day. Pathological examination of the specimens revealed atrophic testes without malignancy. No hernia recurrence was observed during follow-up. The laparoscopic approach in the combined pathology of inguinal hernia and cryptorchidism is feasible in adult patients and has multiple advantages in terms of diagnosis and management.

## Introduction

Cryptorchidism, also known as undescended testis or ectopic testicle, is common in childhood but is rare in adult patients. In the first case, surgical treatment consists of corrective surgery (orchidopexy), whereas in second, orchidectomy is the treatment of choice [1, 2]. This is due to the increased risk of malignancy in adults and the associated low fertility rate associated [3, 4].

Regarding the hernia repair, it is stated today that the laparoscopic techniques have the advantages of less postoperative pain and significantly faster return-to-normal domestic activities and to-work compared to open hernia repair [5, 6].

The common laparoscopic techniques used are total extra-peritoneal (TEP) and trans-abdominal preperitoneal (TAPP) repair, implying good anatomical visualization and easy access to posterior defects. Both techniques imply inserting a mesh in the pre-peritoneal plane, under general anesthesia. In TAPP a laparoscopy is performed whereas in TEP, the peritoneum is left intact. TEP has the advantage of not accessing the peritoneum, whereas TAPP can lead to bowel adhesions to sutures or meshes [7]. Both guidelines describe no difference between TEP and TAPP regarding outcomes [5, 6]. Operative experience is an important factor of decision between these two procedures; nevertheless, HerniaSurge recommends that both techniques are suited for treatment of inguinal hernia [5].

Herein, we report three cases of cryptorchidism associated with inguinal hernia that were treated simultaneously via a laparoscopic approach. Informed consent was obtained from all patients, and the option for orchiectomy was thoroughly discussed before surgery. In all cases, we opted for orchidectomy and TAPP repair of inguinal hernia.

Our aim is to emphasize the advantage of the minimally-invasive approach in the association of cryptorchidism with inguinal hernia and the postoperative outcome.

## Case series

### Case 1

**Figure 1:**
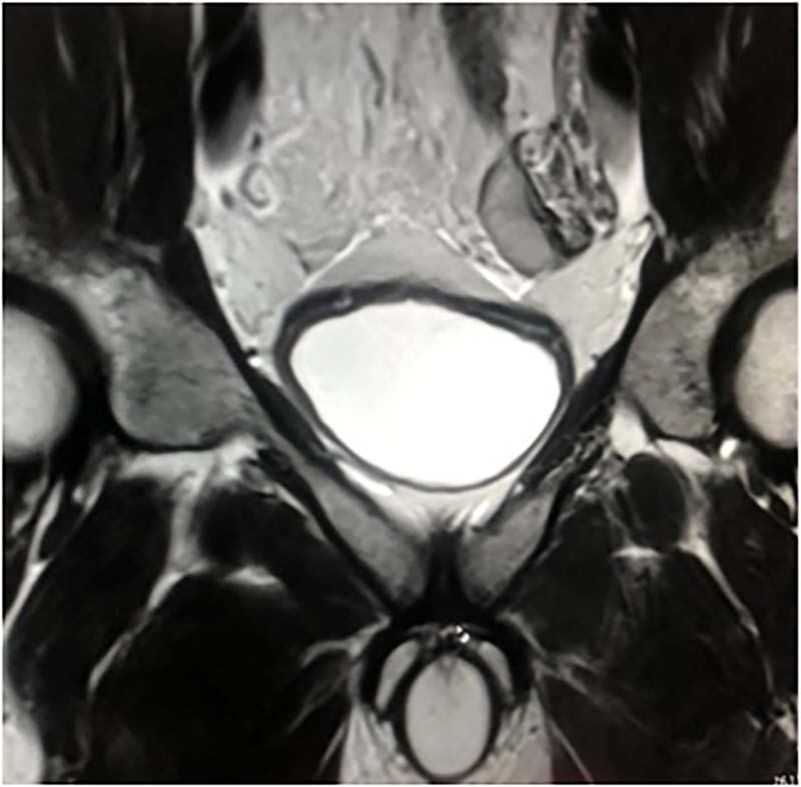
MRI of the case. Left testicle is located in the abdominal cavity.

The first case was that of a 28-year-old-man who was admitted to our hospital with left groin pain for 2 months. Physical examination revealed a left groin bulge and an empty left scrotum. Magnetic resonance imaging (MRI) revealed an undescended testis in the abdominal cavity (Fig. 1). The diagnosis was left inguinal hernia associated with cryptorchidism. Sperm count revealed low fertility.

After discussion with the patient, we opted for an orchidectomy and hernia repair. Subsequently, a laparoscopic approach was performed. Surgery was performed under general anesthesia using a three-trocar setup through the abdominal wall. First, at the umbilicus, and after pneumoperitoneum was created, two other 5 mm trocars were inserted into the right and left middle abdomen. Intraoperative findings revealed a left indirect inguinal hernia, with an orifice diameter of 15 mm.

**Figure 2:**
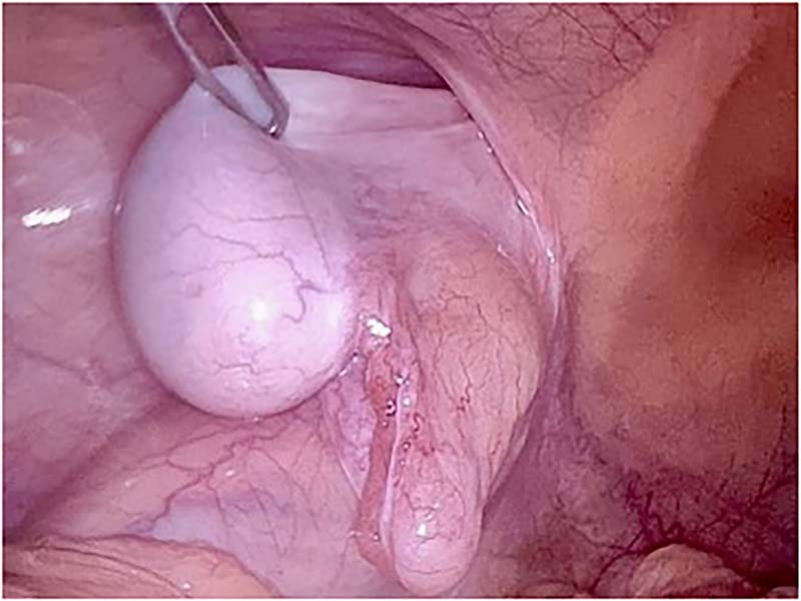
Laparoscopic view of the intraabdominal testicle and the inguinal hernia associated.

**Figure 3:**
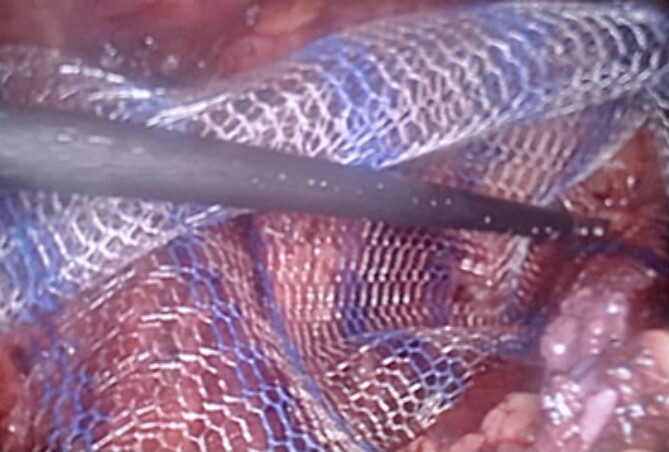
TAPP procedure – lightweight mesh used for the hernia repair.

We also identified the intra-abdominal testis, along with the spermatic cord and testicular vessels (Fig. 2). The right side was normal. Orchidectomy was performed using LigaSure. Testes were extracted through the umbilical port site. After the orchidectomy was performed, left hernioplasty using a lightweight mesh was performed via the TAPP procedure (Fig. 3). The operative time was 75 min. The postoperative course was uneventful, and the patient was discharged on postoperative day. The specimen revealed an atrophic testis without malignancy. Postoperatively, semen analysis revealed improvement, and after 2 years, the patient was married and had two children. To date, there has been no recurrence of the hernia.

### Case 2

A 24-year-old man was admitted with right inguinal bulge since the past 2 months. Physical examination confirmed a right inguinal hernia and empty right side of the scrotum.

Ultrasonography revealed a right inguinal hernia combined with a right atrophic testis in the inguinal canal. The patient was diagnosed with right inguinal hernia associated with cryptorchidism. The patient underwent laparoscopic TAPP repair and orchiectomy. The operative procedure was similar to that in Case 1.

**Figure 4:**
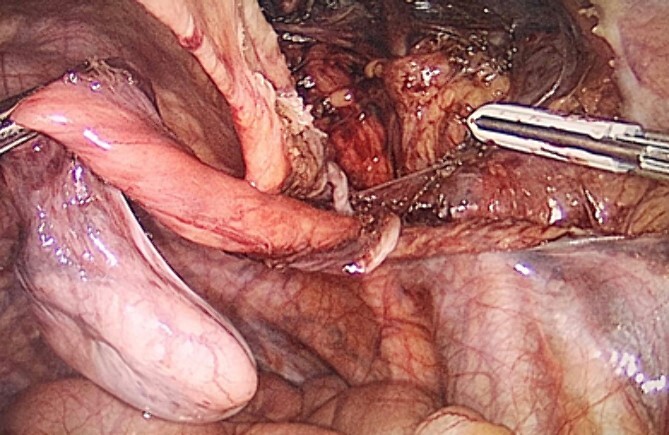
Laparoscopic view of the dissected testicle from the inguinal canal and the hernia sac associated.

**Figure 5:**
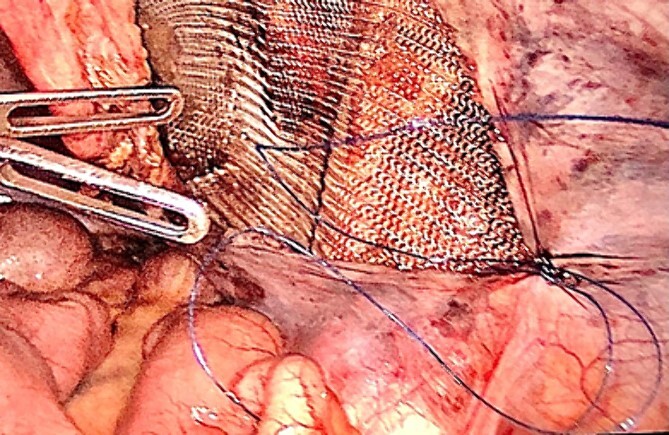
Laparoscopic view of the peritoneal suture in the TAPP procedure performed after orchiectomy.

The hernia sac was found to invaginate the right inguinal ring, and after drawing back the hernia sac, the atrophic testis, along with the spermatic cord and testicular vessels, was revealed (Fig. 4). Heavyweight mesh was used (Fig. 5). The operative time was 95 min. No complications were noted and the patient was discharged on postoperative day 2. Pathological examination revealed no malignancy or spermatogenic abilities. No improvement in the semen analysis results was noted during the follow-up period. To date, there has been no recurrence of the hernia.

### Case 3

A 34-year-old man with left inguinal pain since the past 3 weeks was admitted to our hospital. Physical examination revealed a left inguinal hernia with an empty left scrotum. CT confirmed a left inguinal hernia associated with the left testis located in the abdomen. The patient underwent laparoscopic TAPP repair and orchiectomy. The operative procedure was similar to that in Case 1. The operative time was 102 minutes. No complications were noted and the patient was discharged on postoperative day 2. Pathological examination revealed no malignancy or spermatogenic abilities. No improvement in semen analysis was noted during the follow-up period, and there has been no recurrence of hernia to date.

## Discussion

Cryptorchidism is considered an undescended testis, retractile testis, extra-canalicular testis, intra-canalicular testis, ectopic testis, or intra-abdominal testis [8].

Cryptorchidism, a congenital abnormality, is reported to occur in approximately 1% of children at the age of one year. The incidence of inguinal hernia associated with cryptorchidism is 7% at this age [9]. Moreover, the association of cryptorchidism with hernia is a common finding in childhood and can also be found in adults [8]. Untreated cryptorchid testes are thought to lead to impaired spermatogenesis, one reason for which is that germ cells inside the undescended testis deteriorate after the first year [10]. Moreover, the undescended testis after post-pubertal age remains nonfunctional, and the fertility rate remains low even if orchidopexy is performed [11].

Owing to the low fertility rate and the risk of malignancy, orchidectomy is the recommended procedure for adults [10]. There are reports of testicular carcinoma arising from cryptorchidism in adults [9, 11, 12]. In our patients, a preoperative sperm count revealed impaired semen quality.

Patency of the vaginal process in patients diagnosed with cryptorchidism is associated with the presence of inguinal hernias. The age of presentation for cryptorchidism combined with inguinal hernia is variable, ranging from young adults to the elderly, and has also been reported in a 50-year-old male [13] and even at the age of 84 years [14]. Cryptorchidism related to this late presentation is associated with neglecting the diagnosis or not consulting a pediatrician when an empty scrotum is noted [15].

Hernia associated with cryptorchidism has been identified in many cases during laparoscopy or MRI, but clinically, it can be missed [16]. Ultrasonography, MRI, and CT are useful for identifying and locating the undescended testis. When these findings are negative, laparoscopy plays an important role in cryptorchidism diagnosis and management. In children, many authors consider the laparoscopic approach to be the gold standard [17]. This indication can be extended to adults with this pathology as reported by Vijjan *et al*. [4].

Cryptorchidism is associated with testicular cancer [11], infertility [3], inguinal hernia, and testicular torsion [2]. Interestingly, in rare circumstances, an irreducible hernia can result in cryptorchid testes in the inguinal canal. Sepulveda *et al.* reported a rare case of an adult male [18].

The undescended testis is reported to be intracanalicular or intra-abdominal. The most common location was intracanalicular or close to the internal inguinal ring. The abdominal presence increases the risk of malignancy [15]. An analysis of the pathological reports of testicular specimens of boys with cryptorchidism aged 10 years or above showed malignant changes in 12.5% of boys with intra-abdominal testes, but not in boys with extra-abdominal testes. The authors concluded that orchiectomy or biopsy is necessary for older children with intra-abdominal testes [19]. The specimens in all cases revealed no malignancy. In our study, we found two cases of intra-abdominal cryptorchidism (close to the internal ring) and one case of intra-canalicular cryptorchidism.

The consensus on the management of cryptorchism in adults is controversial; it has been difficult to establish a treatment, given the small number of patients [20]. Nevertheless, some authors favor orchidopexy in selected patients and state that patient preference is important to avoid the negative perception of an empty scrotum [21]. Wood *et al.* suggests that orchiectomy may be considered in healthy patients with cryptorchidism who were between 12 and 50 years [9].

Laparoscopic repair of groin hernias is considered the standard of care, especially for bilateral groin hernias [17, 18]. As stated previously, the laparoscopic approach has several advantages over open surgery, including less wound pain, surgical site occurrence, and superior early discharge. In addition, the detection of hernias (ipsilateral or contralateral) and the possibility of repairing hernias are superior [5, 6].

In adults, the association between cryptorchidism and hernia is uncommon. Regarding the minimally invasive approach for hernia associated with cryptorchidism, some authors favor TEP [8, 22], whereas others TAPP [11, 15, 23, 24]. In a study of 35 patients, aged 26–38 years, Rangarajan *et al*. [15] revealed nine inguinal hernias (25.71%), which were repaired simultaneously with orchidectomy via laparoscopic TAPP procedure. In another report of 14 patients with a mean age of 21 years, 4 (28.57%) had coexistent cryptorchidism and hernia, which were also treated with laparoscopic repair (TAPP) [4].

The mean operative time in our cases was 90.67 min. The postoperative course was uneventful, and two patients were discharged on the first day and one patient on second postoperative day. Postoperatively, one patient showed an improvement in sperm count, whereas the other two did not. No hernia recurrence was noted at 1-year follow-up.

## Conclusions

The association between cryptorchidism and inguinal hernias in adults is rare. Our study presents three cases of cryptorchidism associated with inguinal hernia that were successfully managed with laparoscopic orchiectomy and TAPP hernia repair. No malignancy was detected, and no recurrence of hernia was found during follow-up. The laparoscopic approach is feasible for the treatment of patients with this pathology, and simultaneous treatment has clear advantages. The specimen must be analyzed for the presence of malignant cells.

## Data Availability

Data available upon request.
